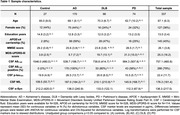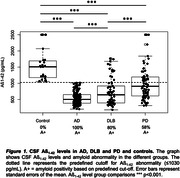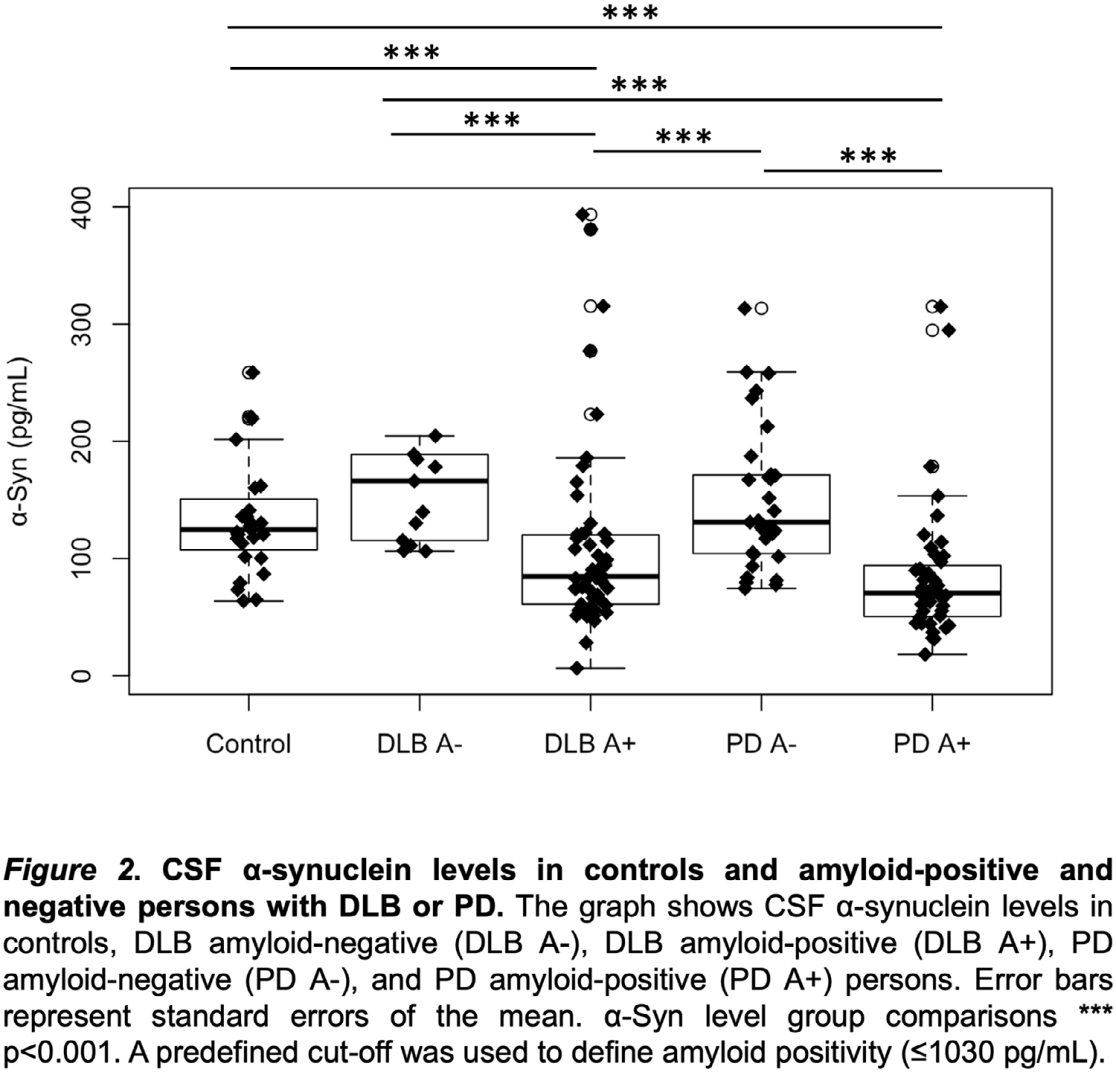# CSF ATN and α‐synuclein co‐pathology in AD, PD, and DLB

**DOI:** 10.1002/alz.086864

**Published:** 2025-01-09

**Authors:** Marianna Rizzo, Charlotte Teunissen, Henrik Zetterberg, Kaj Blennow, Gwendlyn Kollmorgen, Nancy Ramia, Rejko Krüger, Olga Borejko, Dag Aarsland, Michele Hu, Thomas Klockgether, Kathrin Brockmann, Thomas Gasser, Frank Jessen, Annika Spottke, Wiesje M. van der Flier, Afina Willemina Lemstra, Betty M. Tijms, Giovanni B. Frisoni, Jakub Hort, Zuzana Nedelska, Claire Chevalier, Rajaraman Krishnan, Pieter Jelle Visser, Stephanie J. B. Vos, the IMI EPND consortium

**Affiliations:** ^1^ Alzheimer Center Limburg, School for Mental Health and Neuroscience, Maastricht University, Maastricht Netherlands; ^2^ Neurochemistry Laboratory, Department of Clinical Chemistry, Amsterdam Neuroscience, Vrije Universiteit Amsterdam, Amsterdam UMC, Amsterdam Netherlands; ^3^ Clinical Neurochemistry Laboratory, Sahlgrenska University Hospital, Mölndal Sweden; ^4^ Department of Psychiatry and Neurochemistry, Institute of Neuroscience and Physiology, the Sahlgrenska Academy at the University of Gothenburg, Mölndal Sweden; ^5^ Department of Neurodegenerative Disease, UCL Institute of Neurology, London United Kingdom; ^6^ UK Dementia Research Institute at UCL, London United Kingdom; ^7^ Hong Kong Center for Neurodegenerative Diseases, Hong Kong China; ^8^ Wisconsin Alzheimer's Disease Research Center, University of Wisconsin School of Medicine and Public Health, Madison, WI USA; ^9^ Roche Diagnostics GmbH, Penzberg Germany; ^10^ Luxembourg Centre for Systems Biomedicine, University of Luxembourg, Esch‐sur‐Alzette Luxembourg; ^11^ Parkinson Research Clinic, Centre Hospitalier de Luxembourg, Luxembourg Luxembourg; ^12^ Transversal Translational Medicine, Luxembourg Institute of Health, Strassen Luxembourg; ^13^ Institute of Psychiatry, Psychology and Neuroscience, King's College London, London United Kingdom; ^14^ Centre for Age‐Related Medicine, Stavanger University Hospital, Stavanger, Stavanger Norway; ^15^ Nuffield Department of Clinical Neurosciences, University of Oxford, Oxford United Kingdom; ^16^ Oxford Parkinson’s Disease Centre, University of Oxford, Oxford United Kingdom; ^17^ Department of Neurology, University of Bonn, Bonn Germany; ^18^ German Center for Neurodegenerative Diseases (DZNE), Bonn Germany; ^19^ German Center for Neurodegenerative Diseases (DZNE), Tübingen Germany; ^20^ Hertie Institute for Clinical Brain Research, Department of Neurodegenerative Diseases, University of Tübingen, Tübingen Germany; ^21^ Department of Psychiatry, Medical Faculty, University of Cologne, Cologne Germany; ^22^ Alzheimer Center Amsterdam, Department of Neurology, Amsterdam Neuroscience, Vrije Universiteit Amsterdam, Amsterdam UMC, Amsterdam Netherlands; ^23^ Department of Epidemiology and Data Science, Vrije Universiteit Amsterdam, Amsterdam UMC, Amsterdam Netherlands; ^24^ Amsterdam Neuroscience, Neurodegeneration, Amsterdam Netherlands; ^25^ Memory Clinic, Geneva University Hospitals, Geneva Switzerland; ^26^ Laboratory of Neuroimaging of Aging, University of Geneva, Geneva Switzerland; ^27^ Memory Clinic, Department of Neurology, 2nd Faculty of Medicine, Charles University and Motol University Hospital, Prague Czech Republic; ^28^ Sanofi ‐ Rare and Neurologic Diseases Therapeutic Area, Cambridge, MA USA; ^29^ Department of Neurobiology, Care Sciences and Society, Division of Neurogeriatrics, Karolinska Institutet, Stockholm Sweden

## Abstract

**Background:**

Co‐pathology between Alzheimer’s disease (AD), Parkinson’s disease (PD), and dementia with Lewy bodies (DLB) remains poorly understood but is relevant for trial design. We aimed to compare CSF markers of amyloid, tau, and neurodegeneration (ATN) and α‐synuclein between AD, PD, DLB and controls, and investigate the influence of demographical, genetic, and clinical factors on amyloid positivity.

**Method:**

As part of the EPND study, we included 337 individuals with AD, PD, DLB and controls from 6 centers. CSF Aß_1‐42_, p‐tau_181_, NfL, and α‐Syn were centrally measured using NeuroToolKit (Roche Diagnostics International Ltd), and clinical data were harmonized. Controls were individuals with normal cognition and normal Aß_1‐42_. AD was defined as abnormal Aß_1‐42_ without meeting clinical criteria of DLB or PD. Data were analyzed by general linear models adjusted for age and sex.

**Result:**

38% individuals were female, and mean age was 67 years. 170 individuals had AD, 74 PD, 66 DLB, and 27 were controls (Table 1). AD individuals showed lower Aß_1‐42_ levels compared to all other groups (Figure 1). DLB individuals had lower Aß_1‐42_ levels than those with PD, with both groups displaying lower levels than controls. P‐tau_181_ concentrations were higher in AD relative to DLB and PD (both p<0.001), with DLB showing higher (p=0.013) and PD lower (p=0.007) levels than controls. Relative to controls, α‐Syn levels were lower in AD, DLB and PD (p=0.016, p=0.016, and p=0.019, respectively). NfL levels were higher in AD (p=0.003) and DLB (p=0.009) compared to controls. 80% of DLB and 58% of PD individuals were amyloid‐positive. Compared to amyloid‐negative ones, amyloid‐positive DLB and PD individuals had lower α‐Syn levels (both p<0.001, Figure 2). Amyloid‐positive PD individuals had lower p‐tau_181_ levels compared to amyloid‐negative ones (p<0.001). No differences were observed between amyloid‐positive and negative DLB and PD individuals regarding NfL levels. Age, sex, APOE‐ε4, MMSE, and MDS‐UPDRS‐III score were not related to amyloid positivity in DLB and PD.

**Conclusion:**

Amyloid pathology is highly prevalent in DLB and PD and associated with lower α‐synuclein levels. α‐Synuclein levels are also decreased in AD. This highlights overlapping pathologies in AD, DLB and PD and has significant implications for clinical trial designs.